# Competition and cooperation of assembly sequences in recurrent neural networks

**DOI:** 10.1371/journal.pcbi.1013403

**Published:** 2025-09-12

**Authors:** Tristan Manfred Stöber, Andrew B. Lehr, Arash Nikzad, Mohammad Ganjtabesh, Marianne Fyhn, Arvind Kumar

**Affiliations:** 1 Institute for Neural Computation, Ruhr University Bochum, Bochum, Germany; 2 Centre for Integrative Neuroplasticity, University of Oslo, Oslo, Norway; 3 Goethe University Frankfurt, Epilepsy Center Frankfurt Rhine-Main, Department of Neurology, University Medical Center Frankfurt, Frankfurt, Germany; 4 Department of Neuro- and Sensory Physiology, University Medical Center Göttingen, Göttingen, Germany; 5 Department of Computer Science, School of Mathematics, Statistics and Computer Science, College of Science, University of Tehran, Tehran, Iran; 6 Department of Biosciences, University of Oslo, Oslo, Norway; 7 School of Electrical Engineering and Computer Science, KTH Royal Institute of Technology, Stockholm, Sweden; 8 Science For Life Laboratory, Solna, Sweden; École Normale Supérieure, College de France, CNRS, FRANCE

## Abstract

Neural activity sequences are ubiquitous in the brain and play pivotal roles in functions such as long-term memory formation and motor control. While conditions for storing and reactivating individual sequences have been thoroughly characterized, it remains unclear how multiple sequences may interact when activated simultaneously in recurrent neural networks. This question is especially relevant for weak sequences, comprised of fewer neurons, competing against strong sequences. Using a non-linear rate -based and a spiking model with discrete, pre-configured assemblies, we demonstrate that weak sequences can compensate for their competitive disadvantage either by increasing excitatory connections between subsequent assemblies or by cooperating with other co-active sequences. Further, our models suggest that such cooperation can negatively affect sequence speed unless subsequently active assemblies are paired. Our analysis characterizes the conditions for successful sequence progression in isolated, competing, and cooperating assembly sequences, and identifies the distinct contributions of recurrent and feed-forward projections. This proof-of-principle study shows how even disadvantaged sequences can be prioritized for reactivation, a process which has recently been implicated in hippocampal memory processing.

## Author summary

While competition and cooperation are well studied phenomena in various domains of life, the conditions under which they occur in neuronal circuits require further investigation. A central open question is what allows a neural activity sequence to reactivate when competing sequences are present. To address this knowledge gap, we built two types of simple network models: rate-based models and more biological-plausible spiking models. Each model contained up to three sequences, composed of discrete neuronal assemblies connected by feed-forward and recurrent projections. We found that a) both feed-forward and recurrent projections play crucial roles for a sequence to withstand competition, b) weak sequences can overcome their competitive disadvantage through cooperation, c) Hebbian cooperation can however negatively affect sequence speed, d) unless cooperative projections are shifted forward in time. Despite their simplicity, our rate-based and spiking models allow us to make predictions on network properties and plasticity mechanisms in brain regions involved in sequence processing. Given the ubiquitous role of neural activity sequences for information processing, a deeper comprehension of their dynamics will help to unravel basic and pathological brain functions.

## 1. Introduction

Sequences of neural activity are a universal phenomenon in the brain, fundamentally underpinning a range of functions including olfactory processing [20], birdsong generation [23], motor control [15], and episodic memory encoding in the hippocampus [11,13,19,47]. These sequences unfold over various timescales and can be driven by either external stimuli or intrinsic mechanisms. Thus, to understand information processing in the brain, we need to comprehend the dynamics of neural activity sequences.

The emergence and reliable propagation of individual neural activity sequences have been extensively studied using computational models [1,2,4,5,8,12,18,26,28,30,32,35,38–40,43,46,53,57,58,69]. A number of studies characterized conditions for storing and reactivating multiple sequences in recurrent networks [1,4,5,32,35,51,57]. However, interactions between sequences within a network are less understood, and in particular the influence of competition and cooperation on sequence reactivation has not yet come into focus.

A popular experimental paradigm to expose the functional role of neural activity sequences is to record the activity of hippocampal neurons in a spatial navigation task, commonly performed in rats or mice. While traversing an environment, place cells are activated in a sequential manner [13,47]. Subsequently, when the animal is resting, planning or consuming, the same neural activity sequences may be reactivated (or replayed) at a faster time scale during sharp wave ripple (SWR) events [11,29,56,66]. Such offline reactivation can represent multiple distinct experiences [54]. However, it is assumed that normally only one sequence is reactivated per sharp-wave ripple [24].

Replay of sequences is crucial for memory consolidation [14,17,21,48]. Successful generation of long sequences during SWR is associated with better memory [17]. Moreover, the probability that a particular sequence will be reactivated varies with experience, with novel and reward-related sequences being prioritized [3,27,42,55, but see [22]]. Intriguingly, the fact that neither the generation of sharp-wave ripples [6,68] nor the reactivation of sequences [7] are abolished by lesions or inhibition of the medial entorhinal cortex, the primary input structure to the hippocampus, suggests the existence of inherent mechanisms for sequence prioritization within the hippocampus.

Hippocampal activity sequences differ in key properties depending on which information they represent. When encoding the location of objects and other animals fewer hippocampal place cells are recruited and their firing rates are lower compared to place cells for the animal’s own location [9,49]. Thus, hippocampal sequences are likely composed of differently sized cell assemblies. In the following we call sequences with large assemblies strong and those with small assemblies weak. To consolidate their corresponding experiences, it is conceivable that both weak and strong sequences compete for reactivation during SWRs.

A computational model suggests that successful reactivation becomes more difficult for weak sequences, unless recurrent connections within and/or feed-forward projections between cell assemblies are strengthened [8]. However, the required amount of potentiation increases non-linearly with decreasing assembly size, and synapses may quickly reach their physiological boundaries [8]. If multiple sequences are activated at the same time, mutual inhibition between them may create a winner-take-all type competition. In such a scenario, weak sequences essentially stand no chance of winning the competition.

Here, we explore how weak sequences may cooperate to win over stronger sequences during replay events. Inspired by recent findings about gated synaptic plasticity and mutual feed-forward inhibition between region CA3 and CA2 in the hippocampus, we proposed that co-occurring sequences in these regions may be selectively paired by the release of neuromodulatory substances [59]. In addition to linking distinct information [34,41,67] in each region, mutual excitatory support between CA3 and CA2 sequences may ensure their reactivation, while at the same time recruiting sufficient inhibition to suppress competing sequences [24,36].

To develop a theoretical understanding based on these hippocampal insights, we demonstrate that cooperation and competition of assembly sequences can be implemented both in rate-based and spiking models. Within a sequence, reliable and fast signal transmission is achieved by excitatory feed-forward projections between subsequent assemblies, employing balanced amplification [8,45]. Competition and cooperation are implemented by feed-forward inhibition and excitation across assemblies. Characterizing conditions for competition and cooperation, we show that a) feed-forward excitation is crucial, but must remain within a certain range to avoid excessive and persistent activation, b) recurrence within assemblies helps the surviving sequence to recover, c) feed-forward inhibition can mediate competition, d) excitatory coupling between co-active assemblies allows weak sequences to win, but slows sequence progression, and e) preferentially pairing subsequently instead of co-active assemblies maintains sequence speed. Taken together, these results demonstrate that reactivation dynamics of neural sequences are shaped both by modifying feed-forward properties as well as by interactions among multiple sequences.

## 2. Results

### 2.1. Conditions for progression of a single sequence

**Fig 1:**
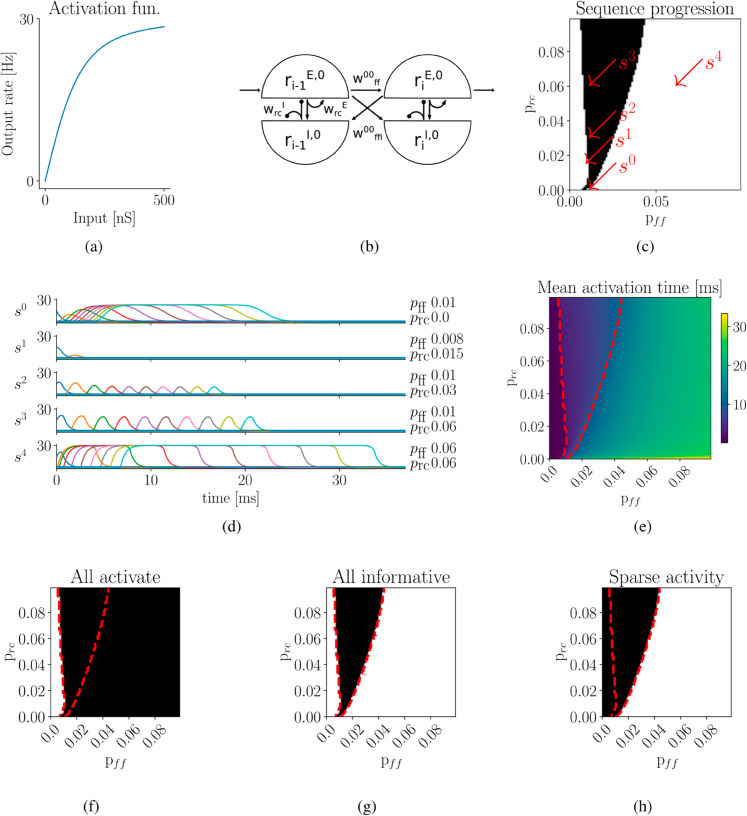
Recurrent and feed-forward interactions influence the progression of a single sequence. **a)** Non-linear activation function of excitatory and inhibitory populations. **b)** Connections within and between assemblies in a single sequence. Each assembly is formed by two recurrently interacting populations, representing excitatory (E) and inhibitory (I) neurons. A given sequence *s*^0^ is established by connecting subsequent excitatory populations via feed-forward excitatory projections with strength $w_{ff}^{00}$. Assemblies generally suppress each other via feed-forward inhibition; excitatory projections from excitatory to all inhibitory populations of other assemblies with strength $w_{ffi}^{00}$. **c)** Successful sequence progression, black region, depends on both recurrent, *p*_*rc*_ and, feed-forward, *p*_*ff*_, connection probability. Red line, analytic solution of the linearized rate model for sustained activity propagation. For low values of *p*_*rc*_, the non-linear rate model and the analytic solution diverge. **d)** Example sequences, corresponding to red arrows in b). Only *s*^2^ and *s*^3^ successfully reactivate. Activity $r_{i}^{E,j}$ of every third excitatory population is shown. Different colors correspond to different excitatory populations. **e)** Mean activation time across all assemblies. Large values of *p*_*ff*_ lead to persistent activity and, thus, to large mean activation time. **f)** Parameter region (black) where all excitatory populations in sequence become activated, fulfilling condition 1: *All active*. **g)** Parameter region fulfilling condition 2: *All informative*, all excitatory populations must exceed activity of others at least once (black). **h)** Parameter region fulfilling condition 3: *Sparse activity*. Red, dashed contours in c), e), f) and g) correspond to black region for successful sequence progression in b).

We used a rate-based model with a non-linear activation function to first study the progression of a single assembly sequence (Fig 1a, 1b). Each assembly is composed of discrete and recurrently interacting populations of excitatory and inhibitory neurons. Sequences are defined by connecting subsequent excitatory populations with feed-forward projections. In addition, all assemblies – independent of their position in the sequence – send feed-forward inhibition to each other; they send excitatory projections to each other’s inhibitory populations. Parameter values are chosen to be in approximate agreement with experimental findings and existing computational models (see Table 2). For example, the activation function reflects documented peak rates of pyramidal neurons in the hippocampus both in vivo [41] and in vitro [16], and timescale *τ* has been adjusted such that individual assemblies in the single sequence case are active for around 5ms, which approximates activation times in a spiking model of assembly sequences [8]. Please note that the time constant can be flexibly adjusted to represent activation times or sequence speeds found in real brains.

To characterize successful sequence progression, we defined four conditions: 1) All active: Within each assembly, the excitatory population must be activated at least at one point in time. 2) All informative: In addition, each excitatory population must exceed the activity of others at least one point in time. 3) Sparse activity: Global activity of the whole network must be sparse, e.g. peak activity is not to be reached by more than two assemblies at any point in time. 4) Order: Peak activation of any excitatory population must maintain its predefined order.

Successful sequence progression depends on both recurrent and feed-forward projections. The strength of each connection is a product of the respective excitatory or inhibitory population size, *M*^{*E*,*I*}^, synaptic strength, *g*^{*E*,*I*}^ and recurrent or feed-forward connection probability, *p*_{*rc*,*ff*}_. To investigate the dependence of sequence progression on connection strength, we systematically varied *p*_*rc*_ and *p*_*ff*_, simultaneously for excitatory and inhibitory projections. We found that the parameter region allowing successful sequence progression for *p*_*ff*_ is relatively narrow compared to *p*_*rc*_ (Fig 1c, black region). Closer investigation revealed that, without sufficient feed-forward projections, activity dies out (*s*^1^ in Fig 1d), preventing all assemblies from being activated (Fig 1f), violating condition 1 (Fig 1g). By contrast, strong feed-forward projections led to rapid and persistent activation (*s*^4^ in Fig 1d, 1e), violating the condition 2 (Fig 1h). However, if excitatory and inhibitory populations recurrently interact with sufficient strength, assembly activation can become transient, allowing sequences to progress in a sparse fashion for an increasing range of feed-forward weights, condition 3 (*s*^2^ and *s*^3^ in Fig 1d).

The V-shape of the parameter region reflecting successful progression illustrates the dual role of recurrent interaction (see Fig 1c). On its left flank (for weak feed-forward connections), increasing recurrent interactions, *p*_*rc*_>0.025, decreases the required feed-forward weights, *p*_*ff*_ by positively amplifying weak inputs. On the right flank (for strong feed-forward connections), stronger recurrent inhibition prevents persistent activity and, thus, increases permissible feed-forward weights. For extremely low recurrence values, $p_{rc}\sim 0$, sequence progression is limited to few specific values of *p*_*ff*_ (*s*^0^ in Fig 1d, 1e).

### 2.2 Competition between two sequences

**Fig 2:**
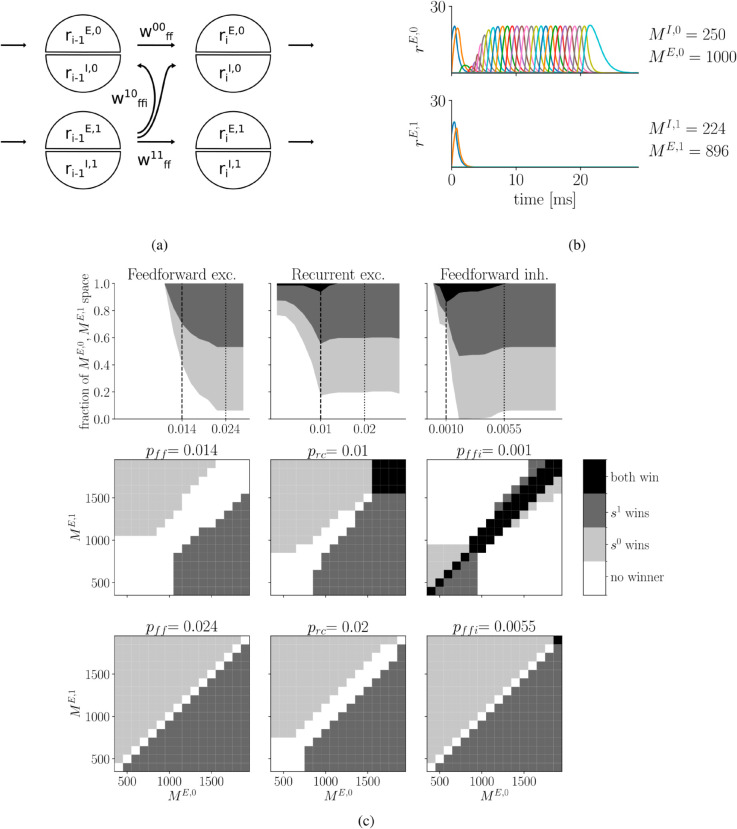
Competition between two sequences. **a)** Scheme of connections for competition scenario. Two sequences, *s*^0^ and *s*^1^, compete via feed-forward inhibition between all assemblies. For visual clarity, only feed-forward inhibition with strength $w_{ffi}^{01}$ from one assembly of *s*^0^ to two assemblies of *s*^1^ is shown. **b)** Example: Larger sequence *s*^0^ wins over *s*^1^. Only activities of excitatory populations are shown. Both sequences suppress each other’s activity until *s*^1^ ceases and *s*^0^ recovers. The last assembly of *s*^1^ maintains its activity for longer because there is no subsequently active assembly from which it may receive inhibition. Colors repeat after 10 assemblies. **c)** Competition scenarios for different values of feed-forward, recurrent, and feed-forward inhibition connection probability. Each column represents a separate parameter scan for feed-forward *p*_*ff*_, recurrent *p*_*rc*_, feed-forward inhibition *p*_*ffi*_ connection probabilities. For each value of the respective connection probability we make a full scan across the *M*^*E*,0^,*M*^*E*,1^ space. The upper row summarizes the fraction of the *M*^*E*,0^,*M*^*E*,1^ space covered by the four possible outcomes: both win, black; *s*^1^ wins, dark grey, *s*^0^ wins, light grey, and no winner, white. Middle and bottom rows provide examples of the *M*^*E*,0^,*M*^*E*,1^ space for different values of *p*_*ff*_, left, *p*_*rc*_, center, or *p*_*ffi*_, right, as indicated by the dashed and pointed lines. Note, origin in plots in b) and upper row in c) at (0,0).

Next, we studied competition between two sequences, *s*^0^ and *s*^1^. As before, each assembly sends feed-forward inhibition to all other assemblies, both within and between sequences (Fig 2a). If the first assemblies in both sequences are simultaneously activated, the interplay between excitation within the assemblies and inhibition between sequences can lead to one of four scenarios: a) Activity in both sequences ceases before the sequence is completed; referred to as *no winner*; b) *s*^0^ successfully progresses and *s*^1^ ceases; referred to as *s*^*0*^ *wins*; c) *s*^1^ successfully progresses and *s*^0^ ceases; referred to as *s*^*1*^ *wins*; d) both sequences successfully progress; referred to as *both win*.

To exemplify competition dynamics, we let two sequences compete for a given set of parameters (Fig 2b). After an initial surge, activity diminished in both sequences. While *s*^1^ ceased, activity in *s*^0^, with slightly larger assemblies, recovered and successfully progressed.

The competition outcome depends on assembly sizes as well as interactions within and between sequences. To systematically characterize the occurrence of the four competition scenarios, we varied assembly sizes *M*^*E*,0^, *M*^*E*,1^ for different connection probabilities of either feed-forward excitation *p*_*ff*_, recurrent excitation *p*_*rc*_, or feed-forward inhibition *p*_*ffi*_. Note, in the following, sizes of the inhibitory populations, *M*^*I*,0^, *M*^*I*,1^, are scaled accordingly to maintain a constant ratio of excitatory and inhibitory population sizes.

For example, for moderate levels of feed-forward excitation, *p*_*ff*_ = 0.014, relatively large assemblies, *M*^*E*,0^, *M*^*E*,1^>1400 were required for one sequence to win over the other (Fig 2c, central row in left column). Nevertheless, even for large assemblies, the difference between sequences had to be prominent, otherwise both sequences ceased to exist. By contrast, if feed-forward excitation is increased, even moderately sized assembly sequences could win as long as they are larger than their competitor (Fig 2c, bottom row in left column). As a consequence of this, the fraction of the *M*^*E*,0^, *M*^*E*,1^ parameter space spanned by either *s*^0^ or *s*^1^ winning increased with a rise in the strength of feed-forward connections, *p*_*ff*_, until it hit an upper bound (Fig 2c, upper row in left column).

Without recurrence, even sequences with large assemblies failed to successfully propagate when competing. As we showed in Figure 1c and know from the literature on synfire chains [12,26,33], individual sequences can progress without recurrent interactions. However, we hypothesized that in a competition scenario, recurrence is paramount for the surviving sequence to recover. To test this, we characterized the competition outcome for a range of assembly sizes given different values of *p*_*rc*_. Consistent with our expectation, for relatively weak recurrence, *p*_*rc*_ = 0.01, larger assemblies were required to avoid that both sequences cease their progression (Fig 2c, central row in central column). Surprisingly, we found that weak recurrence allows both strong sequences to win (Fig 2c, black region, central row in central column). With an increase in the recurrence, *p*_*rc*_ = 0.02, the fraction of the *M*^*E*,0^,*M*^*E*,1^ parameter space spanned by either *s*^0^ or *s*^1^ winning increased (Fig 2c, bottom row in central column). Thus, we conclude that recurrence is indeed crucial when sequences compete.

Feed-forward inhibition ensures that only one sequence wins. Here, sequences competed by inhibiting each other. Therefore, we expected that relatively weak feed-forward inhibition will allow both sequences to win. Again classifying competitions outcomes, we could indeed show that for low values of *p*_*ffi*_ a considerable fraction of the *M*^*E*,0^,*M*^*E*,1^ parameter space was covered by the *both win* scenario (Fig 2c, black region, upper and central row, right column). Further, we found that weak feed-forward inhibition corresponded to a large fraction of failed progressions for both sequences, *no winner*. On the other hand, when *p*_*ffi*_ was increased, the *both win* case disappeared (Fig 2c, upper and bottom row, left column).

**Fig 3:**
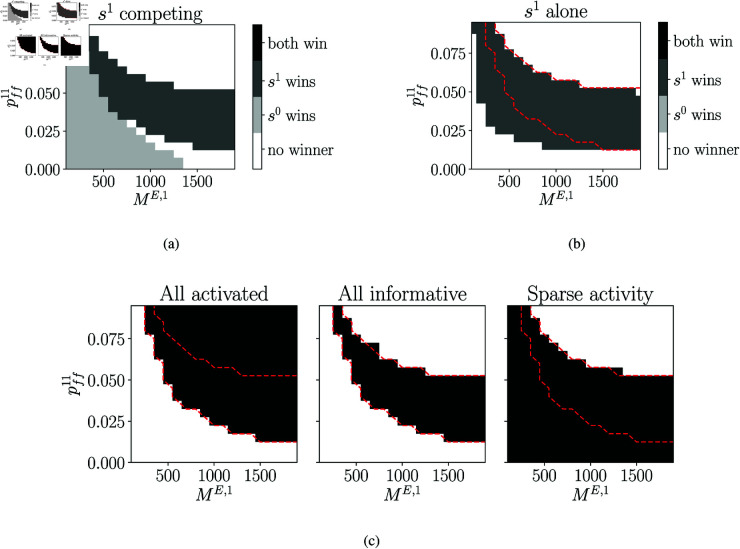
Competition makes it harder for sequences with small assemblies to ensure progression by strengthening feed-forward weights. **a)** For fixed parameters of *s*^0^, *M*^*E*,0^ = 1000, $p_{ff}^{00}=0.02$, assembly size *M*^*E*,1^ and feed-forward connection probability $p_{ff}^{11}$ of *s*^1^ are varied. For *s*^1^ to win (dark area), smaller assemblies must be compensated by increasingly larger feed-forward weights. **b)** Same as a), but without activating *s*^0^.

Competition makes it harder for sequences with small assemblies to ensure progression by strengthening feed-forward projections. As proposed in [59], a weak sequence, comprised of smaller assemblies, competing with a strong sequence, can ensure progression by further potentiating feed forward projections [see also 61]. However, the required strengthening scales non-linearly with assembly size [8] and therefore may hit physiological boundaries for weak sequences. We hypothesized that this becomes even more severe during competition and tested this prediction by varying the respective parameters for sequence *s*^1^, while keeping parameters in *s*^0^ fixed. As expected, we found a non-linear increase in the required feed-forward connection probability $p_{ff}^{11}$ for decreasing assembly sizes *M*^*E*,1^ (Fig 3a). If both assembly sizes and feed-forward weights were strong (*no winner region* in upper right corner of Fig 3a), persisting activity violated both the activation and the sparsity condition (Fig 3c).

To compare the presented results to a situation without competition, we repeated the simulation with silenced *s*^0^ (Fig 3b). As before, the required feed-forward connection probability increased non-linearly with decreasing assembly size. However, without a competing sequence, smaller feed-forward connection probabilities allowed successful propagation. In conclusion, these findings show that competition increases the required strength of feed-forward weights, making it even more difficult to reactivate sequences with small assemblies.

### 2.3 Cooperation and competition between three sequences

**Fig 4:**
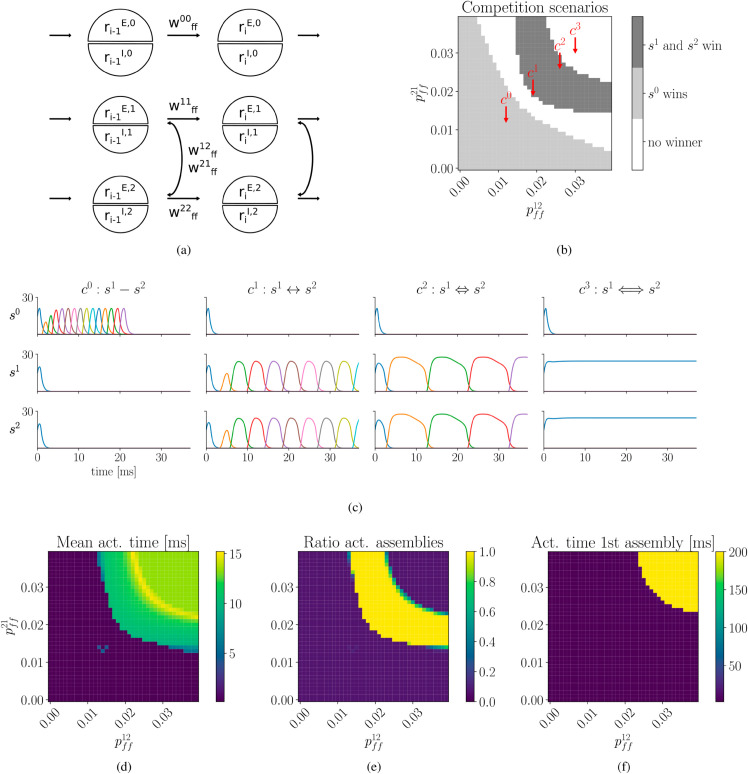
Cooperation via mutual excitation between assembly sequences. **a)** Network scheme for competition and cooperation between discrete assembly sequences. Sequence with larger assemblies, *s*^0^, competes with *s*^1^ and *s*^2^. Competition via feed-forward inhibition between all sequences not shown. Cooperation between *s*^1^ and *s*^2^ through reciprocal excitatory connections to co-active assemblies with strength $w_{ff}^{12}$ and $w_{ff}^{21}$. Larger assemblies of *s*^0^ are indicated by larger circles. **b)** Connection probabilities between *s*^1^ and *s*^2^, $p_{ff}^{12}$ and $p_{ff}^{21}$, are varied. Sufficiently strong mutual excitation is required for *s*^1^ and *s*^2^ to outcompete *s*^0^. **c)** Examples: $c_{0}:s^{1}\;-\;s^{2}$, mutual excitatory interactions between *s*^1^ and *s*^2^ not sufficient; $c_{1}:s^{1}↔s^{2}$, pairing between *s*^1^ and *s*^2^ strong enough to win; $c_{2}:s^{1}\Leftrightarrow s^{2}$, increased excitatory interactions lead to slower sequence progression, e.g. longer activation times; $c_{3}:s^{1}⟺s^{2}$, if excitatory interactions are too strong, sequence progression fails because first assemblies of *s*^1^ and *s*^2^ remain active. Only activity of every second excitatory population is shown. **d)** Mean activation times of excitatory populations in *s*^1^. Strong mutual excitation leads to longer activation times and slow sequence progression. **e)** Ratio of activated excitatory populations in *s*^1^. Only in region with successful cooperation with *s*^2^ all assemblies of *s*^1^ are activated. **f)** Activation time of first excitatory population in *s*^1^. Strong interactions halt propagation, because early assemblies maintain activity over the full simulation duration.

Given the physiological limits on the potentiation of feed-forward projections, an alternative or additional way for sequences to ensure progression despite competition is to mutually support each other. This may happen if simultaneously active assemblies in co-occurring sequences are paired by Hebbian plasticity [59]. To demonstrate both cooperation and competition between assembly sequences, we created a minimal scenario with one strong, and two weak sequences (Fig 4a). As before, all assemblies mutually inhibited each other and the excitatory populations at the start of each sequence were simultaneously activated. The strong sequence *s*^0^ has a competitive advantage due to its larger assemblies. As expected, without any cooperation between *s*^1^ and *s*^2^, sequence *s*^0^ won (case *c*_0_, Fig 4b, 4c).

When weak sequences were able to cooperate, they could however overcome a strong competitor. We introduced feed-forward excitatory projections between co-active excitatory populations in *s*^1^ and *s*^2^, summarized by their strengths $w_{ff}^{12}$ and $w_{ff}^{21}$. Given sufficient mutual support, *s*^1^ and *s*^2^ were able to out-compete *s*^0^ (*c*_1_, Fig 4b, 4c). However, the stronger the mutual excitatory connections, the longer were the activation times of excitatory populations (*c*_1_ vs. *c*_2_, Fig 4d). Increasing the excitatory interactions further led to persistent activity in the first assemblies, halting successful sequence progression (*c*_3_, Fig 4c, 4e, 4f).

### 2.4 Potentiation of excitatory synapses to subsequently active assemblies in paired sequence increases propagation speed

**Fig 5:**
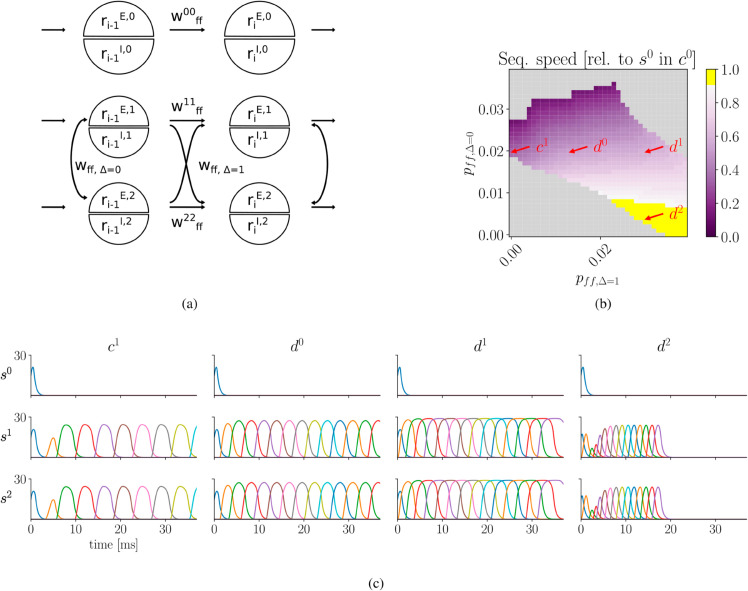
Shifting feedforward excitation from co-active to subsequent assemblies increases speed of cooperating sequences. **a)** Beyond prior simulations which modified feedforward excitation solely between simultaneously active assemblies, $w_{ff,\Delta =0}$, we also introduce feedforward excitation to subsequent assemblies, $w_{ff,\Delta =1}$, in cooperating sequences *s*^1^ and *s*^2^. **b)** Sequence speed of *s*^1^, relative to *s*^0^ of scenario *c*^0^ from Fig 4b. Successful reactivations of *s*^1^ and *s*^2^ are shown for reduced (shades of purple) and similar speed as competing sequence (yellow interval, from 0.9-1.). Non-successful reactivations are indicated in grey. Adjustments in weights $w_{ff,\Delta =0}$ and $w_{ff,\Delta =1}$ are achieved by altering the corresponding connection probabilities $p_{ff,\Delta =0}$ and $p_{ff,\Delta =1}$. **c)** Examples: *c*^1^ - From Fig 4b without pairing between subsequently active assemblies *d*^0^ - Increasing feedforward excitation to subsequent assemblies can counterbalance the reduced speed brought on by the additional feedforward excitation between co-active assemblies. *d*^1^ - Further increasing feedforward excitation to subsequent assemblies expands activation duration of individual assemblies. *d*^2^ - To reach speed of competing sequence *s*^0^ from scenario *c*^0^, feedforward excitation to co-active assemblies must be reduced.

In the previous section we observed that pairing sequences by potentiating co-active assemblies can indeed facilitate their reactivation, but it slows sequence progression and, if too strong, leads to persistent activity. Thus, we hypothesized that sequence speed can be increased by introducing excitatory projections to subsequently active assemblies (see Fig 5a). Adding this type of projection to the three sequence model and explicitly measuring sequence speed by the inverse of the median interpeak interval of excitatory populations, we observed a range of different speeds depending on the relative levels of potentation between co-active and subsequent assemblies (Fig 5b,5c). As expected, stronger potentiation between co-active assemblies led to slower progression (purple region, Fig 5b). Additionally increasing the synaptic strength between subsequent while maintaining strong synapses between co-active assemblies marginally increased speed at the expense of prolonged activation times of individual assemblies (*c*^0^ vs. *d*^0^ and *d*^1^). However, reducing synapse strength between co-active while maintaining relatively strong synapses to subsequent assemblies can increase sequence speed up to the level of the competing sequence *s*^0^ (yellow region, Fig 5b; example *d*^2^, Fig 5c). Thus, potentiating subsequently active assemblies can indeed facilitate reactivation of paired sequences while preserving the timescale of sequence progression.

### 2.5 Sequence competition and cooperation can be implemented in spiking neural networks

**Fig 6:**
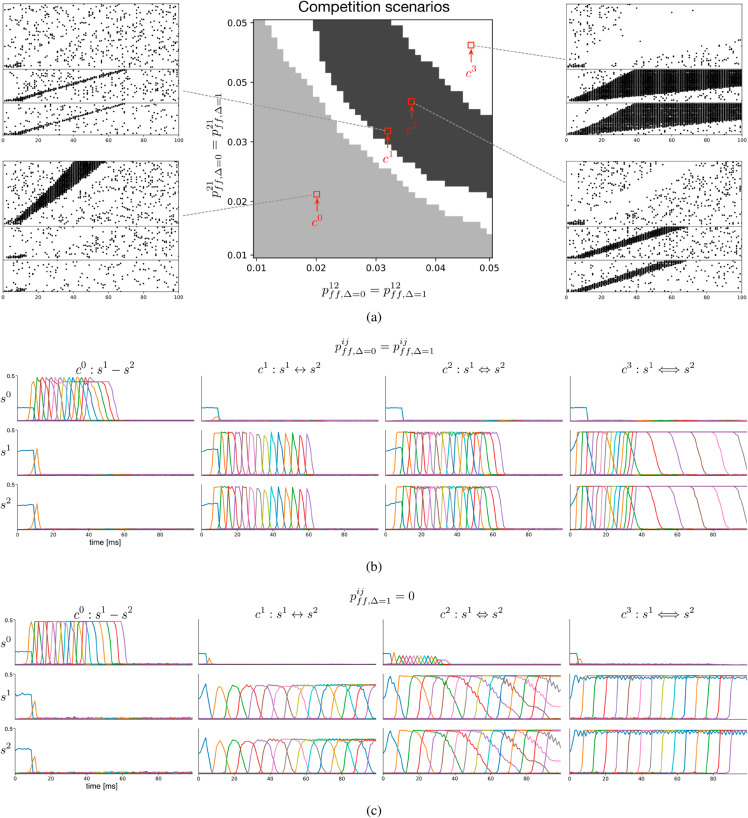
Sequence competition and cooperation can be implemented in spiking neural networks. **a)** Connection probabilities between *s*^1^ and *s*^2^ are varied. This includes both connections between co-active assemblies, as well as those to subsequently active assemblies with, $p_{ff,\Delta =0}^{12}=p_{ff,\Delta =1}^{12}$ and $p_{ff,\Delta =0}^{21}=p_{ff,\Delta =1}^{21}$. As before, in order for *s*^1^ and *s*^2^ to outcompete *s*^0^, a sufficiently strong connection is needed (dark grey area). Raster plots exemplify spiking activity of competing sequences. **b)** Averaged population activity of 10 independent simulations, with parameters corresponding to examples in a). $c_{0}:s^{1}-s^{2}$, mutual excitatory interactions between *s*^1^ and *s*^2^ are not sufficient, *s*^0^ wins; $c_{1}:s^{1}↔s^{2}$, pairing between *s*^1^ and *s*^2^ is strong enough to overcome *s*^0^ and succesfully progress. $c_{2}:s^{1}\Leftrightarrow s^{2}$, increased excitatory interactions extend assembly activation times; $c_{3}:s^{1}⟺s^{2}$, if excitatory interactions are too strong, sequence progression fails because assemblies of *s*^1^ and *s*^2^ remain active. **c)** Similar experiments as in b), with the only difference that $p_{ff,\Delta =1}^{ij}=0$. Note the reduced the propagation speed. In b) and c) activity of every second excitatory population is shown, according to the equation 6.

Rate-based models do not fully capture the temporal dynamics and variability inherent to spiking neural networks. Thus, to validate our findings in a more biologically plausible setting, we replicated sequence cooperation and competition in networks of Leaky Integrate-and-Fire (LIF) neurons with current-based synapses. As before, we let a dominant sequence *s*^0^ compete with two weaker sequences *s*^1^ and *s*^2^, characterize the parameter space for interactions between assemblies of the weaker sequences, and outline different cases of successful or unsuccessful cooperation (Fig 6). To avoid sequence slowdown upon pairing, we jointly increased excitatory projections between co-active and subsequently active assemblies of weak sequences, $p_{ff,\Delta =0}=p_{ff,\Delta =1}$.

Similar to our previous results, the strong sequence *s*^0^ reliably outcompeted the weaker sequences *s*^1^ and *s*^2^ - in case the latter two do not sufficiently cooperate (case *c*^0^). However, simultaneously increasing excitatory interactions between co-active and subsequently active assemblies of *s*^1^ and *s*^2^, allowed them to jointly overcome the strong sequence (case *c*^1^ and *c*^2^). As in the rate-based model, we find both a transition region, without a clear winner (Fig 6a, middle white region), as well as a region of sustained activity, which prevented proper sequence progression, (Fig 6a, upper right white region, and case *c*^3^). Thus, even in spiking neural networks, cooperative excitation must remain within certain boundaries to allow for successful sequence propagation. In agreement with the rate model, cooperation wihtout forward-oriented connections $p_{ff,\Delta =1}=0$, drastically slowed sequence progression (Fig 6c).

To enforce sparse activity, we limit the number of neurons allowed to transmit action potentials within each assembly and timestep via a k-Winners-Take-All (kWTA) mechanism. Such a mechanism may be interpreted as a form of inhibition acting on a fast timescale. Alternatively, we demonstrate successful sequence competition and cooperation with LIF neurons with adaptive membrane dynamics, avoiding the kWTA mechanism (see Fig A in S1 Text).

Taken together these results corroborate our findings from the rate-based model with more biologically plausible spiking dynamics.

## 3 Discussion

Using both non-linear rate-based and spiking models with discrete and pre-configured assemblies, we provided a proof-of-principle for competition and cooperation between neural activity sequences. While a simplification of biological complexity, these models allowed us to study the dynamics of isolated, competing and cooperating sequences. Characterizing conditions for successful sequence progression, we can attribute specific roles to the interactions within and between assemblies. Projections between subsequent excitatory populations ensure sequence progression. However, if too weak, activity does not propagate, and if too strong, activity saturates. Recurrent excitatory and inhibitory interactions implement balanced amplification which boosts weak excitatory inputs and prevents saturating activity [25,45]. Thus, with increasing recurrency, a larger range of excitatory inputs is permissible. Further, the boost of weak inputs is especially beneficial in the competition scenario and allows the surviving sequence to quickly recover. Excitatory interactions between co-active assemblies allow weak sequences to win against a stronger competitor, but such interactions slow the propagation of activity. Shifting feedforward excitation from co-active to subsequent assemblies of cooperating sequences increases sequence speed, enabling successful replay without slowing sequence propagation.

Similar dynamics should be present if more than three sequences interact. To be maximally parsimonious on competition and cooperation, this work considers only three cases: one isolated, two competing and two cooperating sequences competing with another sequence. However, if more than two competing sequences are simultaneously activated, we expect qualitatively similar dynamics. Mutual inhibition, while presumably rising faster, may silence all but the strongest sequence, which would recover and successfully reactivate. This model could in principle be extended to allow for more than two sequences to cooperate and here we would also expect similar dynamics as in the two sequence case: Cooperation would increase their chance for successful reactivation.

Our results highlight a key constraint on which synapses may be potentiated to support successful pairing of activity sequences. We report that direct excitatory interactions between co-active assemblies lead to increased activation times and slower sequence propagation. Maintaining propagation speed for paired sequences was made possible by potentiating excitatory projections to subsequently active excitatory populations in the cooperating sequence. Such temporally skewed potentiation may naturally occur via asymmetric spike-timing-dependent plasticity during encoding [31,44]. We note that several other mechanisms may modulate speed: Dynamic firing rate adaptation to mimic refractory periods [65], inhibitory oscillations to rhythmically gate propagation [50], or inhibitory plasticity to maintain EI balance [64].

The presented results equally relate to the creation of new synapses as well as to potentiation of existing synapses. The strength of an individual connection is defined by the product of population size, the average connection probability and the synaptic weight. Unlike population size which also affects other projections of this population, the specific connection probability and synaptic weight are interchangeable scaling factors.

Weak sequences may also compensate for small assembly sizes by potentiating recurrent interactions, weakening feed-forward inhibition, or recruiting more neurons [assembly outgrowth, see 37,60]. Here, the underlying learning scenario is highly simplified. We assume that during learning pre-configured, recurrently interacting assemblies are activated by external input. This is thought to induce the formation or potentiation of excitatory projections between subsequently activated excitatory populations. For this reason we only evaluated the possibility that weak sequences compensate for small assemblies by strengthening projections between subsequent excitatory populations.

Competition between neural activity sequences may be directly observed in hippocampal recordings. If reactivation of neural activity sequences in the hippocampus is indeed the outcome of a competition process, signatures of this process should be detectable. In the presented model, competition dynamics are characterized by an initial rise in the activity of assemblies of different sequences, followed by reduced activity due to mutual inhibition, until one sequence starts to out-compete the others. Such dynamics should be particularly strong if competing sequences are of equal strength. Studying the reactivation of place cell sequences after running on two or more distinct linear tracks may be an adequate experimental paradigm [24,54].

In summary, our work investigated the interaction of multiple sequences of different strengths within both rate-based and spiking recurrent neural networks. We considered scenarios of competition and cooperation between interacting sequences and characterized the effects on sequence reactivation and sequence dynamics. We showed that pairing weak sequences allows them to win over a stronger competitor. This has implications for hippocampal replay – the number of hippocampal neurons recruited to represent certain types of information strongly differ between sensory modalities [9,52], thus making it important to develop a theoretical understanding of how heterogeneity in assembly size influences replay statistics.

## 4 Methods

For a summarized description of all parameters used in the rate-based and the spiking models, see Table 1. For a summary of all used parameters see Table 2.

**Table 1:** Description of parameters in the non-linear rate model and the spiking model.

Parameter	Description
*M* ^*E*,*i*^	number of excitatory neurons per assembly in *s*^*i*^
*M* ^*I*,*i*^	number of inhibitory neurons per assembly in*s*^*i*^
$r_{\text{peak}}$	peak activation rate [Hz]
*τ*	population time constant [ms]
*a*	rightward shift of activation function
*g* ^*E*^	strength of excitatory synapses [nS]
*g* ^*I*^	strength of inhibitory synapses[nS]
*p*_*rc*_	recurrent connection probability
$p_{ff}^{ii}$	feed-forward exc. connection probability between subsequent assemblies in *s*^*i*^
$p_{ff}^{ij}$	feed-forward exc. connection probability between co-active assemblies in *s*^*i*^ and *s*^*j*^
$p_{ff,\Delta =1}^{ij}$	feed-forward exc. connection probability between subsequently active assemblies in *s*^*i*^ and *s*^*j*^
*p*_*ffi*_	feed-forward inhibition connection probability
*n*_*ass*_	number of assemblies per sequence
*r* ^0^	initial activity of first assembly [Hz]
*t*	simulation time [ms]
*r*_*min*_	minimal activity for classification [Hz]
*r*_*tol*_	activity tolerance for classification [Hz]
$\tau_{n}$	time constant of LIF neurons [ms]
$v_{rest}$	resting membrane potential of LIF neurons
$v_{reset}$	reset membrane potential of LIF neurons
*R*	resistance of LIF neuron [Ω]
*θ*	spiking threshold of LIF neurons [mv]
*dt*	time resolution of spiking simulation [ms]
*r*_*bg*_	rate of background activity of each population [Hz]

**Table 2:**
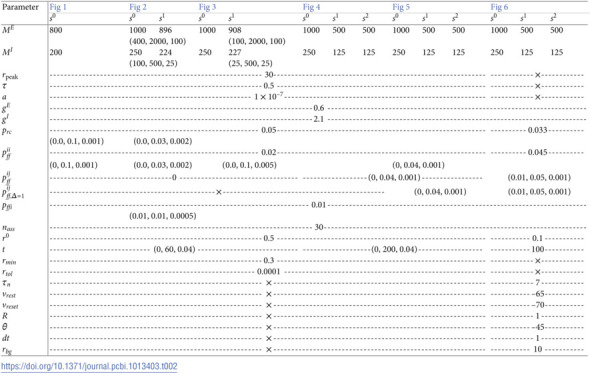
Parameters of the rate-based and the spiking model. Columns are organized in figures and sequences. If necessary, rows are split into default parameters, upper part, and parameter ranges, lower part. If not otherwise stated, default parameters are used. Ranges are indicated with (start, stop, stepsize). Dashed lines indicate values applying to multiple columns. The irrelevant parameters for each experiment are shown by × symbol.

### 4.1. Conditions for successful sequence reactivation

To be classified as successfully progressing, a sequence must satisfy the following four conditions: 1) All active: All assemblies must be activated. There should exist at least one point in time during which the activity of a given excitatory population exceeds a minimal threshold *r*_*min*_. 2) All informative: Each excitatory population must exceed the activity of others at least one point in time. 3) Sparse activity: While the sequence is running, maximum firing rates at any given point in time must not be reached by more than two assemblies simultaneously. To exclude numerical edge cases we consider assemblies to have similar firing rates, whenever the absolute value of the difference is less than *r*_*tol*_. Allowing two assemblies to both have peak activity is necessary for the time points when decreasing activity of the previous and increasing activity of the subsequent assembly are equal. Condition 2 and 3 ensure that each assembly is prominently activated. Without condition 2, a strongly active assembly could overshadow the activation of subsequent assemblies, which would make the readout more challenging. Condition 3 prevents cases where multiple assemblies just saturate at maximum firing rate. 4) Order: Activation times must maintain sequence order. The order of peak activities agrees with the predefined order of assemblies in the sequence. Given our predefined one-step feed-forward interactions this is almost always the case, though we mention it for completeness.

### 4.2. Assembly sequences in a non-linear rate model

In the non-linear rate model each assembly is formed by one excitatory and one inhibitory population. The evolution of rate, $r_{i}^{j}$, of a given population *i* of sequence *s*^*j*^ is described by

$$\tau \frac{dr_{i}^{j}}{dt}=-r_{i}^{j}+S(x)$$ (1)

with *τ* a fixed population time constant, equal across all populations.

The sigmoidal activation function *S* over the input *x* is defined by

$$S(x)=R\left(\frac{\left(x-a\right)}{\sqrt{(x-a{)}^{2}+1}}\right)$$ (2)

with the rectifier R(·)=max(0,·) (compare Fig 1a) and $a=1\times 10^{-7}$ a small constant rightward shift of the activation function, preventing numerical imprecision around *x* = 0 from inadvertently driving network activity.

Each population in sequence *s*^*j*^ receives input by recurrent excitatory and inhibitory projections with strengths $w_{rc}^{E,j}$ and $w_{rc}^{I,j}$. Excitatory populations may receive additional excitatory input by the preceding assembly of the same sequence with strength $w_{ff}^{jj}$. In the case of cooperating sequences, each excitatory population receives excitatory input of a co-active assembly of another sequence *s*^*m*^ with strength $w_{ff}^{mj}$. All assemblies send feed-forward inhibition to each other, e.g. they excite each others inhibitory population. Thus, in addition to the recurrent input $w_{rc}^{E,j}$ from their associated excitatory population, they receive input $w_{ffi}^{mj}$ from all remaining *n* excitatory populations $r_{n}^{E,m}$ of all sequences *s*^*m*^. Thus, the full input to an excitatory population $x_{i}^{E,j}$ and an inhibitory population $x_{i}^{I,j}$ of assembly *i* in sequence *s*^*j*^ is described by:

$$x_{i}^{E,j}=w_{rc}^{E,j}r_{i}^{E,j}+w_{rc}^{I,j}r_{i}^{I,j}+w_{ff}^{jj}r_{i-1}^{E,j}+w_{ff}^{mj}r_{i}^{E,m}$$ (3)

$$x_{i}^{I,j}=w_{rc}^{E,j}r_{i}^{E,j}+w_{rc}^{I,j}r_{i}^{I,j}+\sum_{m}\sum_{n\neq i}w_{ffi}^{mj}r_{n}^{E,m}$$ (4)

Weight values are the product of the number of excitatory, *M*^*E*,*j*^, and inhibitory, *M*^*I*,*j*^, neurons in the sending population, equal for all assemblies in a given sequence *s*^*j*^, as well as recurrent, *p*_*rc*_, feed-forward, *p*_*ff*_, and feed-forward inhibitory, *p*_*ffi*_, connection probabilities and excitatory, *g*^*E*^, or inhibitory, *g*^*I*^, synaptic strengths.

$$w_{rc}^{E,j}=M^{E,j}p_{rc}g^{E}$$ (5)

$$w_{rc}^{I,j}=M^{I,j}p_{rc}g^{I}$$

$$w_{ff}^{mj}=M^{E,m}p_{ff}g^{E}$$

$$w_{ffi}^{mj}=M^{E,m}p_{ffi}g^{E}$$

Sequences are comprised of *n*_*ass*_ assemblies, all assemblies within a given sequence are equal in size and the *E*/*I* size ratio is fixed to $M^{E}/M^{I}=4$.

Sequence speed is determined as the inverse of the median interpeak interval of excitatory populations. Before determining timepoints of peak activation, we rounded values to a precision of *r*_*tol*_ and ignored values below *r*_*min*_ to avoid numerical fluctuations to be considered a peak.

Simulations were run for a fixed time interval and a fixed step size with the solve_ivp function in SciPy’s integrate package with integration method LSODA. As initial condition, the excitatory population in the first assembly of each activated sequence *s*^*j*^ is set to $r_{0}^{E,j}=0.5$, while all other rates are at zero.

Simulations and analysis of rate models were performed with Jupyter notebooks 6.0.3 and Python 3.7.8 with standard libraries, such as NumPy 1.18.5, SciPy 1.18.5, Matplotlib 3.2.2 and SymPy 1.5.1.

### 4.3. Assembly sequences in spiking neural networks

In our experiments, we implemented a network of spiking neurons using PymoNNtorch 0.1.4 [62], a Pytorch-adapted version of PymoNNto [63]. All simulations were executed on CPU. We employed the Leaky Integrate-and-Fire (LIF) neuron model, which is described by the following differential equation:


$$\tau_{n}\frac{dv}{dt}=-(v-v_{rest})+RI(t),$$

where $\tau_{n}$ is the neuronal membrane time constant, $v_{rest}$ represents the resting membrane potential, *R* is the membrane resistance, and *I*(*t*) denotes the synaptic input current received by each neuron. The synaptic input current *I*(*t*) for each neuron is computed as:


$$I_{i}(t)=\sum_{j=1}^{N}W_{ji}\delta (t-t_{j}^{\;\;f}),$$

with *N* the number of pre-synaptic neurons *j* connected to post-synaptic neuron *i*, *W* the connectivity matrix, ($t_{j}^{\;\;f}$) spiking activity of *j*, and *δ* the Dirac delta function, ensuring that input currents are updated at precise spike times.

Each assembly consists of an excitatory and inhibitory population. The ratio of excitatory to inhibitory neurons within each sequence is fixed to 4:1. The simulation duration is set to 100 ms, with a time resolution of 1 ms.

A k-Winners-Take-All (kWTA) mechanism enforces sparse and competitive activation patterns. At any time step, only spikes from the *k* neurons with the highest membrane potential of a population are transmitted. However, membrane potentials of all neurons crossing the firing threshold are reset.

To simulate background activity, at each time step *t*, a uniformly distributed random number $\eta_{i}(t)\sim U(0,1)$ is assigned to each neuron *i*, and those with $\eta_{i}(t)<r$ (where *r* is the predefined activation rate parameter) receive an external input current *I*_*bg*_. This approach mimics the random synaptic inputs observed in biological neural networks, where most neurons receive ongoing background activity even in the absence of specific stimuli [10].

To analyze network dynamics, the average neuronal activity of the excitatory population of each assembly is calculated as:

$$A(t)=\frac{1}{N}\sum_{j=1}^{N}\sum_{f}\delta (t-t_{j}^{\;\;f})$$ (6)

where *A*(*t*) represents the total activity at time *t*, normalized by the number of neurons.

The feedforward and recurrent connectivity schemes were adopted from the previously described rate-based models, ensuring a comparable network structure while incorporating more biologically plausible spiking dynamics. However, for simplicity, feedforward inhibition is limited to co-active assemblies of competing sequences.

To start sequences, we forced the excitatory population of the first assembly to spike for 10 ms. For each time step, we randomly selected 10% of the neurons and injected a current of 1000 mA. The kWTA mechanisms remained active.

## Supporting information

S1 Text Supplementary information Fig A in S1 Text. Sequence competition and cooperation in spiking neural networks with adaptive membrane dynamics.
(PDF)
