# Supplementary material for: Competition and cooperation of assembly sequences in recurrent neural networks
Source: PLoS Comput Biol. 2025 Sep 12;21(9):e1013403. doi: 10.1371/journal.pcbi.1013403 (PMC12469390; doi:10.1371/journal.pcbi.1013403)
Supplement: S1 Text — Sequence competition and cooperation in spiking neural networks with adaptive membrane dynamics. (PDF) [file pcbi.1013403.s001.pdf]

Supplementary Information

Sequence competition and cooperation in spiking neural networks with adaptive membrane dynamics

To demonstrate that sequence competition and cooperation does not strictly depend on the k-Winner-Takes-All mechanism used above, we implemented a version without and use excitatory neurons with simplified adaptive membrane dynamics (Brette & Gerstner, 2003) defined by:

$$\tau_n \frac{dv}{dt} = -(v - v_{rest}) + -R\, w + R\, I(t)$$

$$\tau_w \frac{dw}{dt} = -w + \beta\, \tau_w \sum_t^f \delta(t - t^f)$$

with w the adaptation variable, $\tau_w = 10$ ms the adaptation time constant and $\beta = 20$ describing the spike-triggered adaptation coefficient. All other parameters in the network remain the same as the kWTA version. Inhibitory neurons do not possess adaptive dynamics.

By testing four parameter combinations along, we observe qualitatively similar competition and cooperation dynamics: Only with a certain amount of cooperation, s and s can overcome the stronger sequence s. However, increasing interaction strength leads to longer activation times.

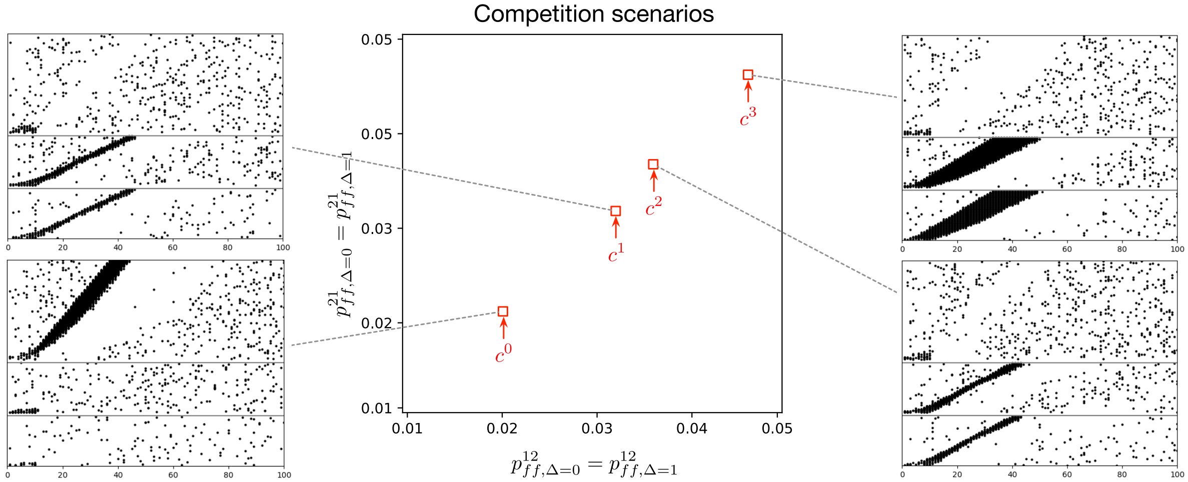

(a)

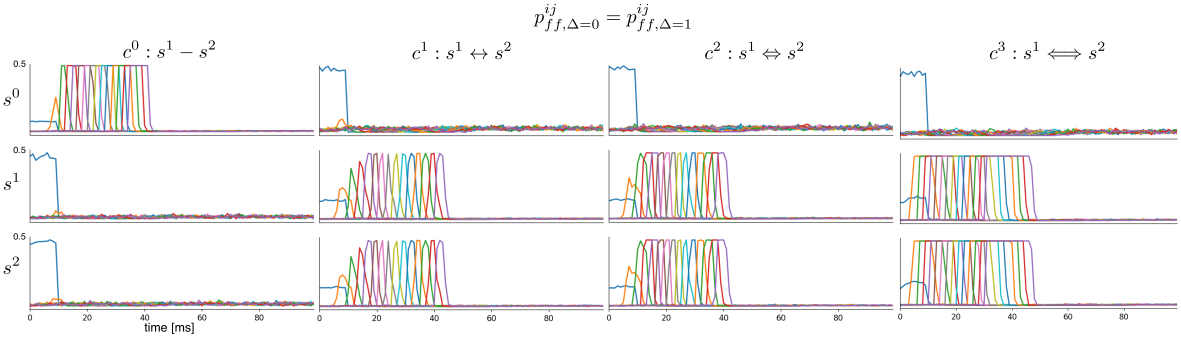

(b)

Fig A. **Sequence competition and cooperation in spiking neural networks with adaptive membrane dynamics. a)** Similar to Fig. **??**, we highlight four parameter combinations, $c - c$, reflecting weak to strong mutual interaction between s and s are displayed with their corresponding raster plots. While no exhaustive search of the parameter space is performed, we observe that in order for s^1 and s^2 to outcompete s^0, a sufficiently strong interaction between s^1 and s^2 is required. **b)** Averaged population activity of 10 independent simulations, with parameters corresponding to examples in a). $c_0 : s^1 - s^2$, mutual excitatory interactions between s^1 and s^2 are not sufficient, s^0 wins; $c_1 : s^1 \leftrightarrow s^2$ and $c_2 : s^1 \Leftrightarrow s^2$, pairing between s^1 and s^2 is strong enough to overcome s^0 and to successfully progress; $c_3 : s^1 \Longleftrightarrow s^2$, increased excitatory interactions lead to long assembly activation times and slow sequence progression. Activity of every second excitatory population is shown, according to the equation **??**.
